# Vasoactive intestinal peptide ameliorates renal injury in a pristane-induced lupus mouse model by modulating Th17/Treg balance

**DOI:** 10.1186/s12882-019-1548-y

**Published:** 2019-09-05

**Authors:** Dongdong Fu, Soulixay Senouthai, Junjie Wang, Yanwu You

**Affiliations:** Department of Nephrology, Affiliated Hospital of Youjiang Medical University for Nationalities, No. 18 Zhongshan Road II, Baise, 533000 Guangxi Zhuang Autonomous Region China

**Keywords:** Vasoactive intestinal peptide, Th17/Treg balance, Lupus nephritis

## Abstract

**Background:**

Lupus nephritis (LN) is an inflammation of the kidneys and is a major cause of mortality in systemic lupus erythaematosus (SLE) patients. In addition, Th17/Treg balance is one of the most important factors that can promote the development of LN. It has been reported that vasoactive intestinal peptide (VIP) is associated with the downregulation of both inflammatory and autoimmune diseases through regulating T lymphocyte balance. Therefore, the aim of this study was to determine the role of VIP in modulating Th17/Treg balance in LN.

**Methods:**

LN was induced in BALB/c female mice by injection pristane. After 3 months, mice were randomly divided into four groups: control, VIP + control, LN and VIP + LN. Autoantibody levels were tested by ELISA. The distribution of Th17/Treg cells in vivo and in vitro was detected by FC. Renal tissues were examined by PASM and DIF for pathology and Foxp3^+^CD3^+^. The mRNA and protein expression levels of pro- and anti-inflammatory cytokines were detected by qRT-PCR and western blotting.

**Results:**

VIP can improve renal injury by regulating Th17/Treg imbalance in LN mice. Proteinuria, renal function defects and autoantibodies were significantly decreased, and Th17/Treg cell balance was restored in VIP compared with LN mice. In addition, VIP improved renal lesions by promoting the expression of Foxp3^+^CD3^+^ in renal tissue. Furthermore, VIP downregulated the mRNA and protein expression of IL-17, IL-6 and upregulated Foxp3, IL-10 expression.

**Conclusions:**

VIP reduced LN proteinuria and renal function defects and restored the Th17/Treg cell balance. Furthermore, VIP also downregulated autoantibody and inflammatory cytokine expression and upregulated Foxp3 and IL-10 expression.

## Background

Lupus nephritis (LN) is a disorder in which the body’s immune system attacks the body’s own cells and organs and is a major cause of mortality in systemic lupus erythaematosus (SLE) patients. Approximately 60% of patients with proliferative SLE progress to end-stage renal disease (ESRD), which affects patient mortality, the kidneys are functioning below 10% of their normal function [1]. The cause of LN is not understood, and multifactorial interactions among various genetic and environmental factors are likely involved. Defective immune regulatory mechanisms, such as the impairment of T cell (Th17/Treg imbalance) function, are important contributors to the development of LN due to proinflammatory cytokine secretion, which greatly stimulates B cells for autoantibody production and contributes to organ damage.

Recently, accumulating evidence has suggested that Th17/Treg cell functional imbalance is closely associated with the pathogenesis of LN [2–4]. Th17 cells play a role in adaptive immunity to protect the body against pathogens, but they have also been implicated in the pathogenesis of tissue inflammation and tissue destruction, which play a critical role in many autoimmune and inflammatory disorders. In contrast, Treg cells can modulate effector T cell function to maintain immunological homeostasis and prevent autoimmunity. Thus, the disruption of Th17/Treg balance is considered the major pathogenic contributor to renal damage in LN. In addition, recovery of the immune balance between Th17 and Treg cells might help to ameliorate disease activity in LN.

Pristane, also known as hydrocarbon oil TMPD (2, 6, 10, 14-tetramethylpentadecane), is capable of inducing a lupus-prone murine model that is similar to human SLE which includes female predominance, nephritis, arthritis and pulmonary vasculitis. In this model, autoantibody production and end-organ damage, including renal disease, depend on the IFN-I receptor signalling pathway. This model is widely used in research to understand the pathogenesis of LN and rheumatoid arthritis [5]. It has been reported that pristane-induced LN mice exhibit immunoregulatory abnormalities in their T cells and B cell hyperreactivity in in vitro immune responses [6].

Vasoactive intestinal peptide (VIP) is a 28-amino acid peptide that is extensively distributed in tissues, which exerts pleiotropic functions in multiple systems, such as the gastrointestinal [7], cardiovascular [8], nervous [9], and immune [10, 11] systems. Many studies have shown that VIP can be used as an immunomodulatory and anti-inflammatory factor that participates in regulating immune balance [10, 12]. It has been reported that VIP can effectively increase the number of Treg subpopulations while decreasing the number of Th17 subpopulations to improve inflammation in asthmatic mice by regulating Th17/Treg balance and VIP can also maintain the immune balance between Th17/Treg cells in the prevention and treatment of experimental autoimmune encephalomyelitis (EAE). Furthermore, VIP can stimulate the proliferation of Treg cells in vitro [13]. However, it remains unclear whether VIP regulates the immune balance of Th17/Treg cells in the pathogenesis of LN.

Therefore, in the present study, we investigated whether VIP might play an important role in the treatment of autoimmune diseases, including LN. To demonstrate the underlying mechanisms of VIP treatment in LN mice, we examined the dynamics of Th17/Treg cells in pristane-induced LN mice and investigated the therapeutic effects of VIP in this model. The present study showed that VIP treatment could effectively ameliorate LN, which might be a consequence of restoring the Th17/Treg balance in pristane-induced LN mice. Collectively, the findings from this study provide new clinical applications for VIP in the treatment of LN.

## Methods

### Reagents

VIP (bs-0077P, Bioss Inc., Beijing) was dissolved in phosphate-buffered saline (PBS, Solabio, Beijing). Pristane (C_19_H_40_) was purchased from Sigma-Aldrich (P2870, St. Louis, MO, USA). Fast Quant RT Super Mix and SYBR Green PCR reagents were purchased for quantitative RT-PCR assays (R6424 and R6306, Tiangen, China). Antibodies for flow cytometry analysis were purchased from BD Biosciences (560,767, San Diego, USA). Enzyme-linked immunosorbent assay (ELISA) kits were purchased from Cusabio Biotech Co., Ltd., China. Flow cytometry detection of Treg and Th17 cell antibodies were from BD Biosciences (BD Biosciences, Cat# 560767, San Diego, USA). CD3 were purchased from Abcam Biotech Co., Ltd., China (ab5690). Foxp3 were purchased from Santa Cruz Biosciences (sc-53,876).

### In vivo studies

#### Mice

Female specific pathogen-free BALB/c mice, aged 8 weeks and weighing 21 ± 3 g, were purchased from Model Animal Research Center of Nanjing University (Nanjing, China). All mice received sterilized food and water indefinitely in an SPF environment and were housed in light and dark within 12:12 h. A maximum of 5 mice per cage. All animal experiments SPF mice were Youjiang Medical Committee for approval, and every effort was made to minimize the damage to mice.

#### Animal experimental design

All female the mice that were reared were divided into four groups (*n* = 5/group): (1) control group, BALB/c mice received a single injection of 0.5 mL normal saline intraperitoneally (i.p.); (2) VIP group, BALB/c mice were treated with 200 μg/mouse VIP (once every 2 days, i.p.); (3) LN group, BALB/c mice received a single injection of 0.5 mL pristane i.p. (Molecular formula: C19H40, Molecular weight: 268.52, density: 0.783) [14]; and (4) LN + VIP group, BALB/c mice were injected with 0.5 ml pristine, and after 3 months, 200 μg/mouse VIP was injected intraperitoneally. After 4 weeks of treatment VIP, the female mice blood samples were collected by tail or retro-orbital puncture (using heparinized glass capillary tubes, EDTAK2) under anesthesia. The spleen and kidney were detached under sterile conditions. Spleen was collected in 1.5 mL tube with RPMI 1640 (1 mL). Renal pole fractions were snap frozen in liquid nitrogen and transversal slices of renal tissue were fixed in 10% formalin.

#### Detection of proteinuria and renal function in mice

Female mice were collected for 24 h in a metabolic cage, peripheral blood was collected from the posterior orbital venous plexus, and serum creatinine (SCr) and blood urea nitrogen (BUN) values were measured after 4 weeks of completion of treatment as described previously [15].

#### ELISA

The serum of each group of mice was taken out from the ultra-low temperature freezer. The concentration of ANA, anti-dsDNA and anti-Sm antibodies in peripheral blood of each mouse was determined using an ELISA kit (Cusabio Biotech Co., Ltd.) in strict accordance with the instructions. Absorbance was measured with a TriStar LB 941 multimode microplate reader (Berthold Technologies, Bad Wildbad, Germany) at 450 nm.

#### Histopathological analysis

To further assess renal pathological changes, right and left kidneys were collected from each of the sacrificed mice and further processed by immersion in 10% neutral buffered formalin and conventional paraffin embedding, and stained with Periodic Acid-silver Methe-namine (PASM) according to standard procedures and examined under a light microscope. The average lesion areas were calculated with ImageJ software. Renal pathology examinations were performed in a blinded manner by a pathologist.

#### Immunofluorescence staining

For double staining with foxp3 and CD3 (for represent Tregs) [16], the sections were carried out using paraffin-embedded sections (4 μm) of renal tissues, and incubated with Foxp3 and CD3 primary antibodies individually overnight at 4 °C. The next day, the samples were incubated with rabbit anti-mouse IgG secondary antibody for 1 h at room temperature. The samples were then observed with a fluorescence microscope (IX71, Olympus, Tokyo, Japan) equipped with ISCapture software, and images were taken with a CCD camera (Discovery C15, Olympus, Tokyo, Japan). The numbers of Foxp3^+^ positive (green) and CD3^+^ positive (red) cells counted under fluorescent microscopy.

### In vitro studies

#### Lymphocyte experimental design

Mice spleen lymphocytes were separated in a sterile environment, adjusted to a cell concentration of 1 × 10^6^/mL with RPMI-1640 medium containing 10% fetal bovine serum (Gibco), and cultured in 6-well plates. Subsequently, the stimulation factors IL-6 (50 ng/mL), TGF-β (1 ng/mL), IL-23 (20 ng/mL), anti-IFNγ (3 μg/mL) and anti-IL-4 (3 μg/mL) were added to induce Th17 cell proliferation, and TGF-β (10 ng/mL) and IL-2 (10 ng/mL) were added to stimulate Treg cell proliferation. After culturing in a 37 °C, 5% CO_2_ incubator for 3 days. The Treg cells were divided randomly into four groups: (1) NC group, Treg cells from BALB/c mice; (2) LN group, Treg cell from pristane-induced lupus mice; (3) VIP group, Treg cells from BALB/c mice were treated with 10^− 7^ M VIP; (4) LN + VIP group, Treg cell from pristane-induced lupus mice were treated with 10^− 7^ M VIP. The Th17 cells were divided randomly into four groups: (1) NC group, Th17 cells from BALB/c mice; (2) LN group, Th17 cell from pristane-induced lupus mice; (3) VIP group, Th17 cells from BALB/c mice were treated with 10^− 7^ M VIP; (4) LN + VIP group, Th17 cell from pristane-induced lupus mice were treated with 10^− 7^ M VIP. The experiment was terminated after 72 h of treatment. The fractions of Treg and Th17 cells in each group were detected by flow cytometry. The mRNA and protein levels of IL-17, IL-6, Foxp3 and IL-10 were detected by qRT-PCR and western blotting.

#### Flow cytometric analysis

The spleen and peripheral blood are collected and the lymphocytes are separated and operated under aseptic conditions. The cell suspension was separated by density gradient centrifugation in mouse lymphocyte isolation medium (Solarbio, Cat# P8860, Beijing, China). The cells of each group were washed three times with sterile PBS buffer. The lymphocyte suspensions (1 × 10^6^/mL) were stained with antibodies specific for mouse CD4, IL-17 (for Th17 cells) and Foxp3 (for Treg cells) or isotype-matched controls according to the manufacturer’s instructions (Cat #560767). The samples were mixed gently, incubated for 30 min at room temperature, and subsequently analysed using a flow cytometer (FC) FACS CantoII (BD Biosciences, San Jose, CA, USA).

### RNA purification and quantitative real-time PCR assays

Renal tissue was collected in RNase-free centrifuge tube using Trizol reagent (lot #103105, Invitrogen) to extract total RNA. For cDNA synthesis, 2 μg purified total RNA was reverse-transcribed (KR116, Tiangen, Beijing, China). The expression levels of IL-17, IL-6, Foxp3, IL-10 and GAPDH were determined using SuperReal PreMix Plus (SYBR Green) (FP205, Tiangen, Beijing) according to the requirements of the manual, the data is used and tested in strict accordance with the specifications. The data was repeated three times for each experiment. The primer sequences are shown in Table 1. Comparative gene expression was calculated by the 2^-ΔΔCt^ method as described previously [17].

**Table 1:** PCR primers used in this study

Gene Name	Forward(5′-3′)	Reverse(5′-3′)
IL-17	TGATGCTGTTGCTGCTGCTGAG	TGGAACGGTTGAGGTAGTCTGAGG
IL-6	AGGAGTGGCTAAGGACCAAGACC	CTGACCACAGTGAGGAATGTCCAC
FOXP3	GAAGAGCCTGCCTTGGTACATTCG	TGTGAAGGTTCCAGTGCTGTTGC
IL-10	CAAGGCAGTGGAGCAGGTGAAG	GCTCTGTCTAGGTCCTGGAGTCC
GAPDH	AACTTTGGCATTGTGGAAGG	GGATGCAGGGATGATGTTCT

### Protein isolation and western blot analysis

The method for protein from total kidney homogenate was extracted according to the manufacturer’s recommended protocol (Cwbiotech, Beijing, China). Proteins were resolved on 10% sodium dodecyl sulfate-polyacrylamide gel electrophoresis (SDS-PAGE) and transferred to nitrocellulose membranes (GE Healthcare, Freiburg, Germany). After transfer, 5% skimmed milk powder was used to block the membranes at room temperature for 1 h. Then, the membranes were pre-incubated with primary Foxp3 (sc-53,876, 1:500 dilution), IL-6 (sc-7920, 1:500 dilution) (Santa Cruz Biotechnology, California, USA), anti-IL-10 (ab9969, 1.5 μg/mL dilution), anti-IL-17 (ab79056, 1.5 μg/mL dilution) (Abcam Ltd., Hongkong, China) and GAPDH (1:1000 dilution) (Danvers, MA, USA) antibodies at 4 °C overnight and then incubated with secondary antibodies following three washes with TBST. Then, to detection of the expression level of the protein of interest using an enhanced chemiluminescence (ECL, Millipore, Billerica, MA, USA) western blot detection kit and a chemiluminescent imaging system (Bio-Rad, USA)..

### Statistical analysis

All data values were mean ± standard error of the mean in the bar graph. Measurements were performed by one-way ANOVA with Newman-Keuls multiple comparison post-test (for more than two comparisons) and paired t-test (comparison of two groups) in data comparison statistical significance analysis. All data were conducted with SPSS 17.0 software (SPSS Inc., Chicago, USA). *P*-values less than 0.05 were regarded significant.

## Results

### An LN mouse model was successfully established

Twelve weeks after pristane injection, significant increases in proteinuria (mg/24 h) (88.17 ± 5.76) and serum Cr (1.39 ± 0.05) and BUN (25.71 ± 4.12) levels were detected in LN mice compared to control mice (25.33 ± 5.48, 0.25 ± 0.05 and 8.51 ± 1.90, respectively, *p* < 0.01). Furthermore, the ANA (63.69 ± 3.56), anti-dsDNA (78.52 ± 4.04) and anti-Sm (51.89 ± 2.17) autoantibodies were significantly higher in LN mice than in control mice (29.42 ± 3.25, 28.58 ± 1.99 and 20.81 ± 3.02, respectively, *p* < 0.01). According to these results, a mouse model of LN was established successfully, and these mice are used in the subsequent studies [18].

### VIP reduces autoantibody levels, proteinuria and renal function defects in LN mice

**Fig. 1:**
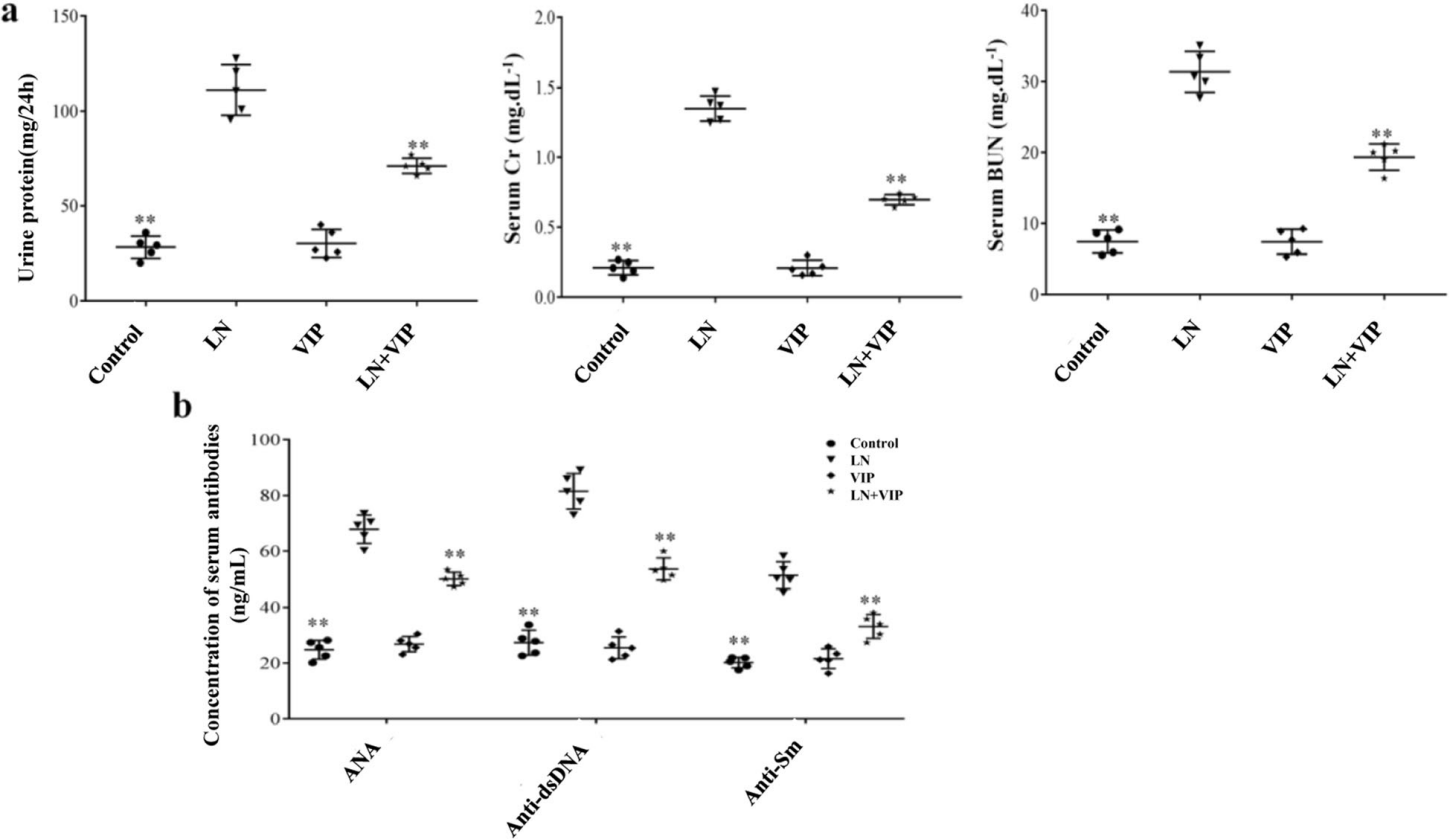
VIP ameliorates the serum levels of autoantibody and renal function in LN mice. After 4 weeks of VIP treatment, urine was collected for 24 h using metabolic cages to determine the protein urine levels, and blood was collected for serum creatinine, BUN and autoantibody measurements. **a** Assessment of proteinuria and kidney function in mice and **b** serum levels of ANA, anti-dsDNA and anti-Sm antibodies. The data are expressed as the mean ± standard deviation (*n* = 5). Statistical analyses were performed using one-way ANOVA. **p < 0.01 compared with LN mice

After 4 weeks of VIP treatment, LN mice presented with significantly less renal injury, as demonstrated by marked reductions in urine protein (mg/24 h), renal function defects and autoantibodies. As shown in Fig. 1, the 24 h proteinuria (111.12 ± 13.28), SCr (1.35 ± 0.09) and BUN (31.36 ± 2.86) levels were higher in LN mice than in LN + VIP mice (71.12 ± 4.02, 0.69 ± 0.36 and 19.36 ± 1.86, respectively, *p* < 0.01) (Fig. 1a). The LN mice also exhibited significantly higher levels of ANA (67.89 ± 5.10), anti-dsDNA (81.52 ± 6.39) and anti-Sm (51.49 ± 4.82) than the LN + VIP-treated mice (51.49 ± 4.82, 53.68 ± 3.90 and 33.16 ± 4.29, respectively, *p* < 0.01) (Fig. 1b). There were no significant differences between the control and VIP mice (*p* > 0.05).

### VIP modulated Th17/Treg imbalance in the peripheral blood and spleens of LN mice

**Fig. 2:**
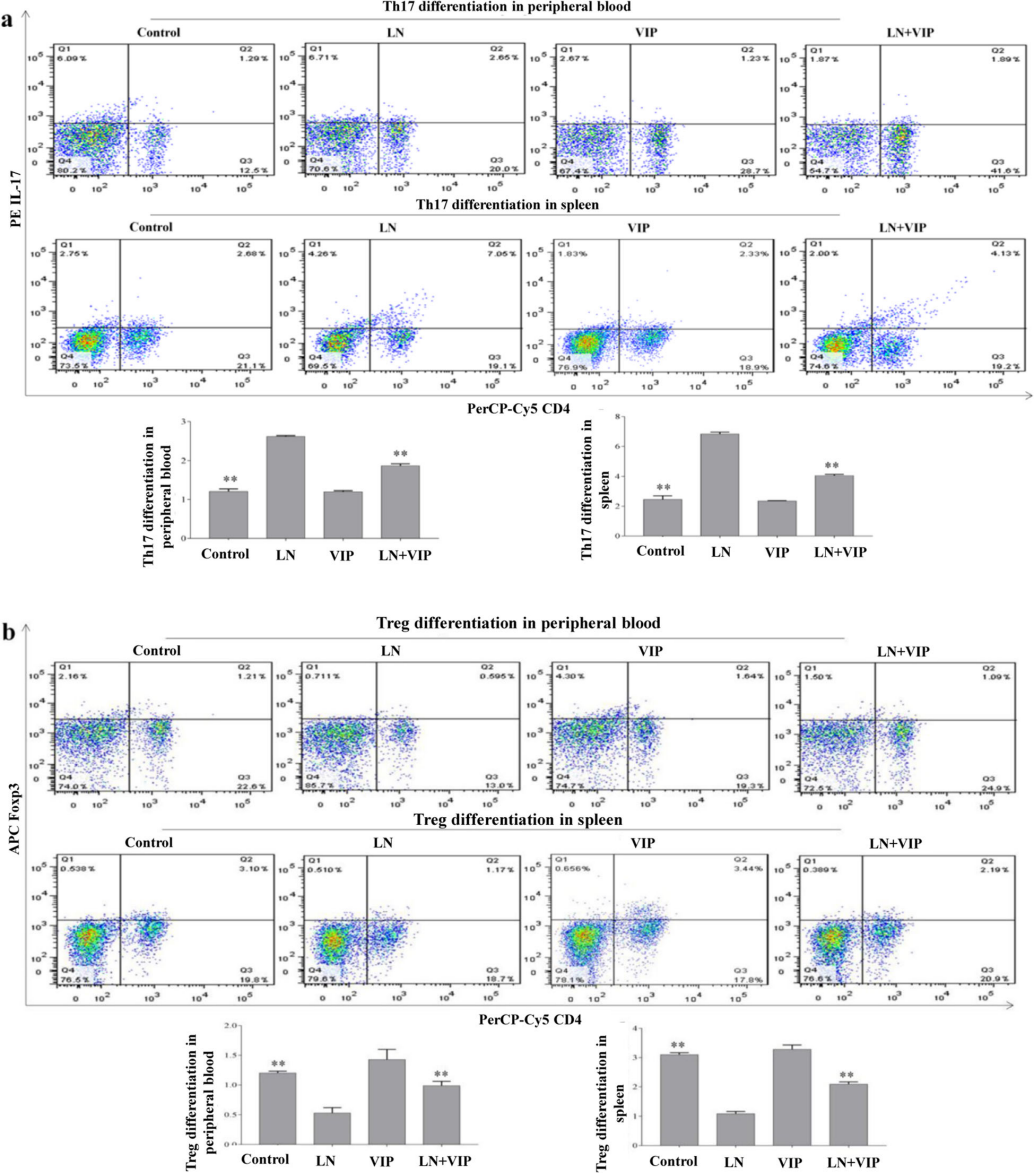
Effects of VIP on modulating the Th17/Treg balance in LN mice. After treatment with VIP for 4 weeks, the peripheral blood and spleens from LN mice were collected and analysed by flow cytometry. The pseudo-coloured flow cytometry picture represents the cellular distribution of one typical sample from each group. **a** Proportion of Th17 cells in the peripheral blood and spleens; **b** proportion of Treg cells in the peripheral blood and spleens. **p < 0.01 compared with LN mice

The number of Th17 cells was significantly increased in the peripheral blood and spleens of LN mice (2.61 ± 0.02 and 6.82 ± 0.13, respectively) (Fig. 2a), while the number of Treg cells was significantly decreased in the peripheral blood and spleens (0.53 ± 0.08 and 1.08 ± 0.07, respectively) (Fig. 2b) when compared with those in the control mice (Th17: 1.21 ± 0.06 and 2.45 ± 0.23 in the peripheral blood and spleen, respectively; Treg: 1.20 ± 0.03 and 3.09 ± 0.07 in the peripheral blood and spleen, respectively, *p* < 0.01). The results also suggest that after LN + VIP treatment, the number of Th17 cells was significantly decreased (1.86 ± 0.05 in the peripheral blood and 4.05 ± 0.07 in the spleen), and the number of Treg cells was significantly increased (0.98 ± 0.07 in the peripheral blood and 2.09 ± 0.07 in the spleen, *p* < 0.01) when compared to those in the LN mice (Fig. 2). However, there were no significant differences between the control and VIP mice (*p* > 0.05). These findings demonstrated that VIP can inhibit Th17 cell differentiation and promote the generation of Treg cells, thereby restoring the Th17/Treg cell balance and ameliorating LN in mice.

### VIP ameliorated the renal lesions in pristane-induced LN mice

**Fig. 3:**
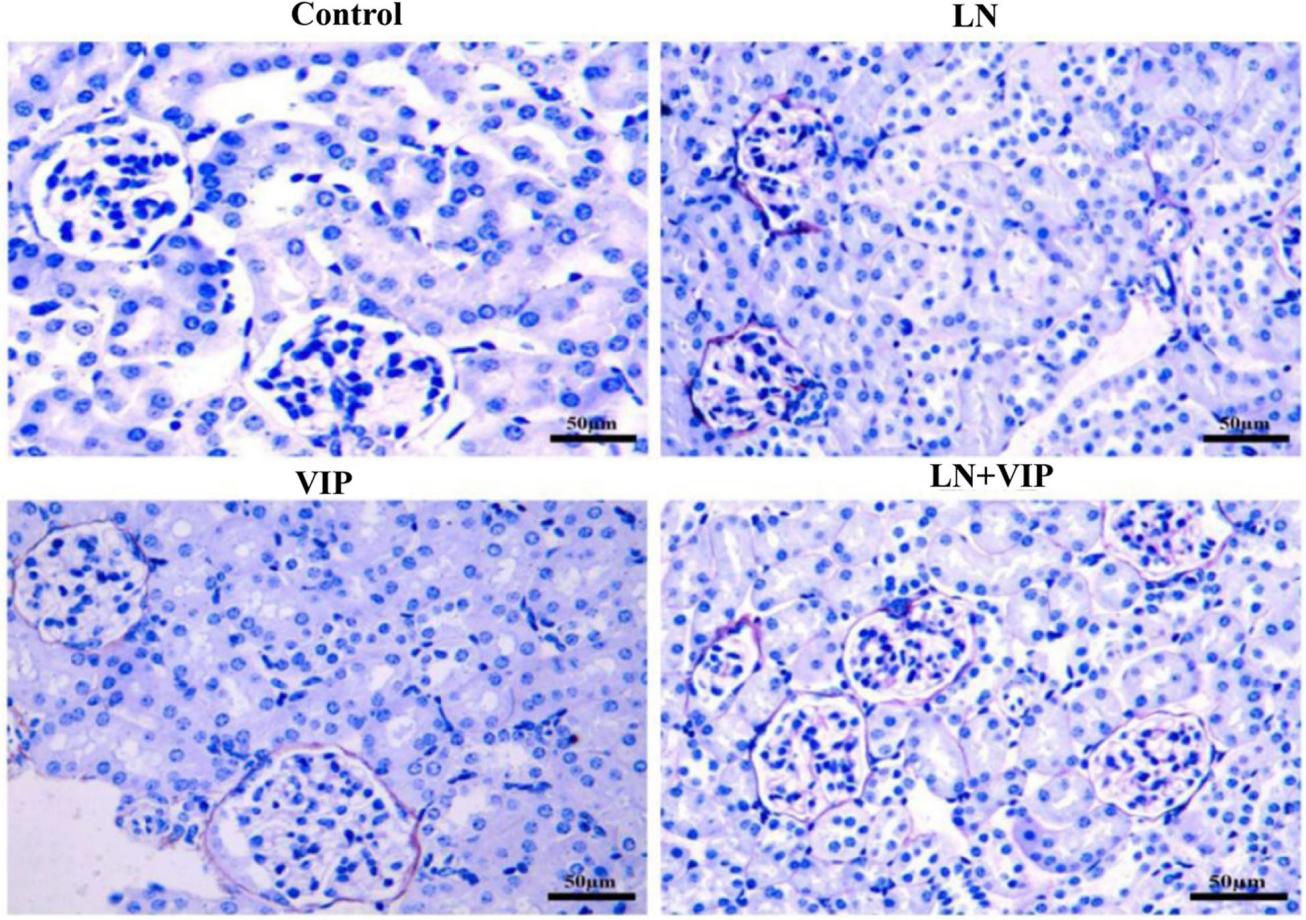
Renal histopathology in LN mice, Histopathological features of the mouse renal tissues were determined by PASM (original magnification, × 400). Histological findings for renal tissues from the four experimental mouse groups: an intact glomerular basement membrane, no hyperaemia, and no necrotic cells were found in the renal tubules of control and VIP mice; LN mice showed significant glomerular atrophy, basement membrane thickening and rupture, mesangial area widening, mesangial matrix increases, mesangial cell proliferation and renal tubule necrosis, suggesting damage to the renal structure; renal cortical pathology in LN + VIP mice revealed less mesangial matrix, decreased mesangial cell proliferation, and decreased basement membrane hyperplasia

The PASM-stained renal tissues in LN mice appeared to have renal glomerular atrophy, basement membrane thickening and rupture, mesangial area widening, mesangial matrix increases, mesangial cell proliferation (more than 55%), and renal tubule necrosis. In the LN + VIP-treated mice, histopathological changes in the renal cortical areas were significantly improved, including less mesangial matrix accumulation and decreased mesangial cell proliferation (less than 25%) (Fig. 3).

### Expression of Foxp3 and CD3 in renal tissue is regulated by VIP

**Fig. 4:**
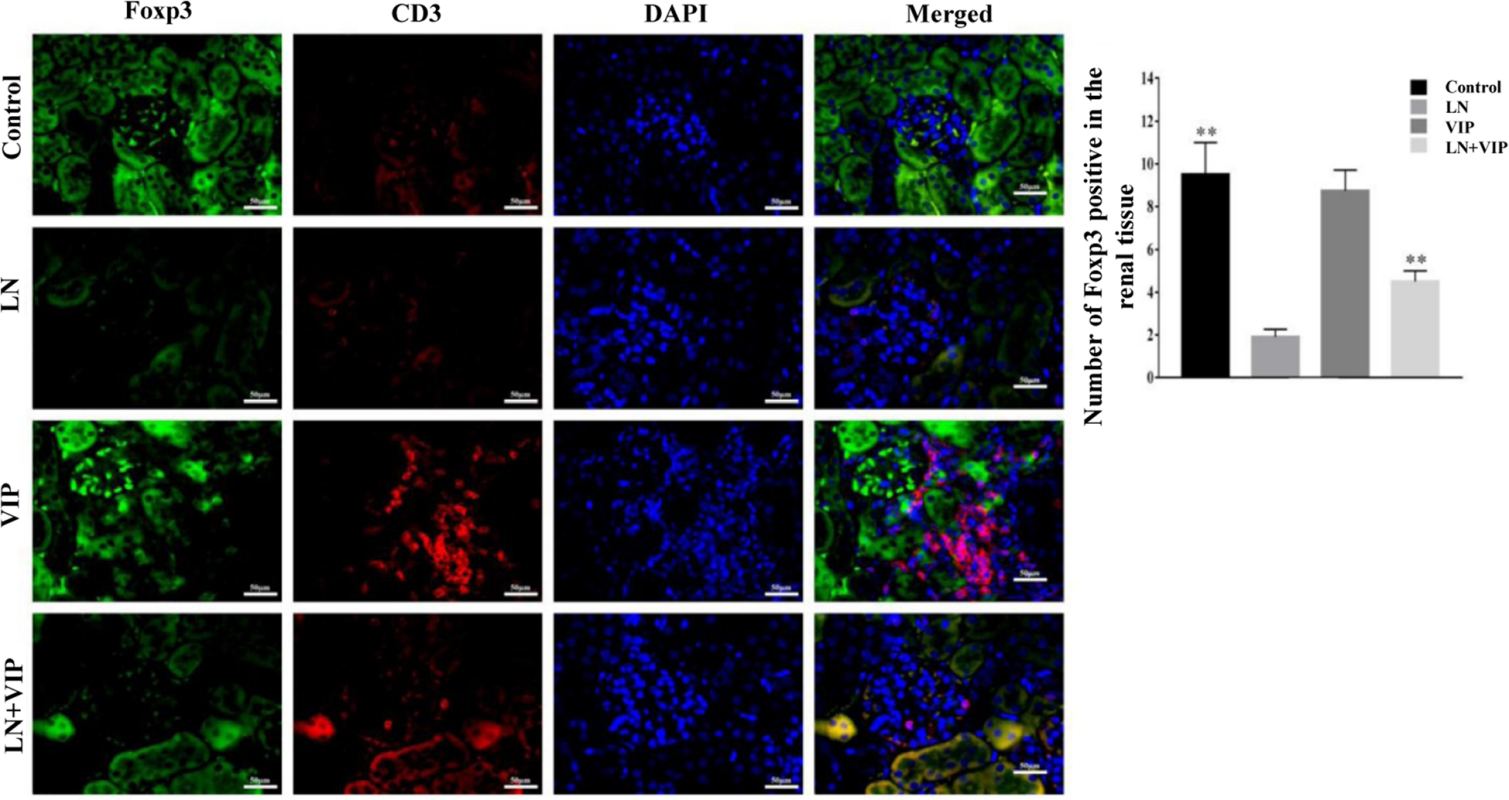
Double immunofluorescence in LN mice. The double immunohistochemical stains (original magnification, × 400) for Foxp3 (green) and CD3 (red) demonstrate a higher number of Foxp3 positive cells in the interstitial cellular infiltrates in the renal tissue of mice. **p < 0.01 compared with LN mice

To determine the therapeutic effect of VIP on renal tissue in LN mice, we analysed kidney sections stained by DIF. VIP obviously improved the pristane-induced renal pathological changes in LN mice. The expression levels of Foxp3 and CD3 compared between the different groups of mice are shown in Fig. 4. According to the DIF staining of renal tissue with antibodies to Foxp3 and CD3 indicated that the expression of Foxp3^+^CD3^+^ were lower in the interstitial cellular infiltrates of LN renal tissue than in the control mice, while the Foxp3^+^CD3^+^ levels were increased after VIP treatment in LN + VIP mice compared to LN mice. These findings indicated that VIP could modulate Th17/Treg immune response and reduced renal injury in LN mice model.

### VIP inhibited Th17 cell differentiation and promoted Treg cell proliferation in lymphocytes from the mouse spleens

**Fig. 5:**
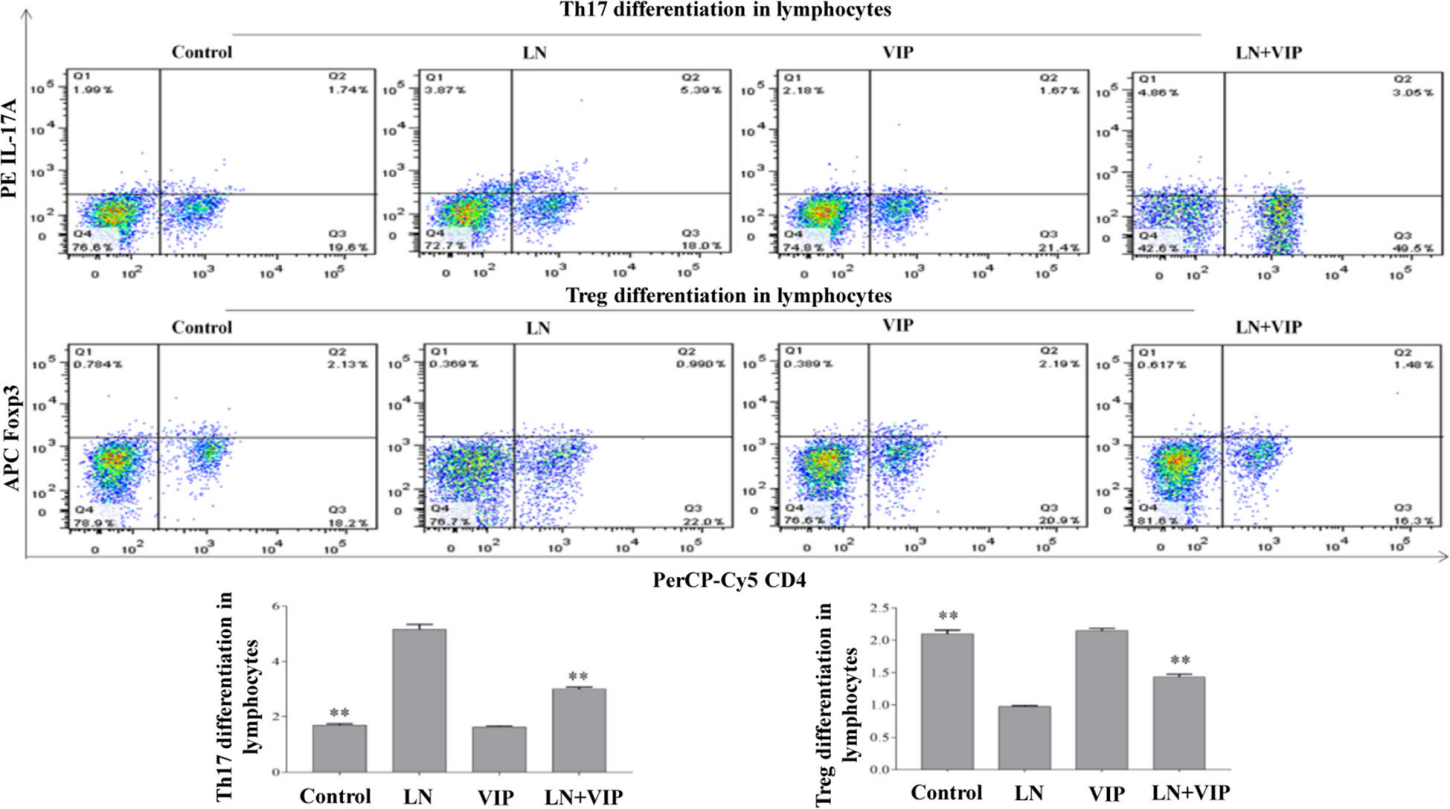
Effects of VIP on the proportion of Th17/Treg cells in lymphocytes in vitro*.* Mouse spleen lymphocytes were separated in a sterile environment, and the experiment was terminated after 72 h of VIP treatment. The fraction of Th17 and Treg cells in each group was detected by flow cytometry. The pseudo-coloured flow cytometry picture represents the cellular distribution of one typical sample from each group. **p < 0.01 compared with LN mice

In this experiment, Th17 and Treg cells in lymphocytes from the spleen were determined by flow cytometric analysis. As shown in Fig. 5, the results show that the LN mice exhibited significantly higher levels of Th17 (5.15 ± 0.18) and lower levels of Treg cells (0.97 ± 0.02) than the control mice (1.69 ± 0.04 and 2.09 ± 0.06, respectively, *p* < 0.01). The number of Th17 cells (2.99 ± 0.07) was significantly lower, and the number of Treg cells (1.43 ± 0.04) was significantly higher in LN + VIP mice than in LN mice (*p* < 0.01). However, there were no significant differences between the control and VIP mice (*p* > 0.05).

### VIP regulated the gene and protein expression of pro- and anti-inflammatory cytokines in the renal tissue of LN mice

**Fig. 6:**
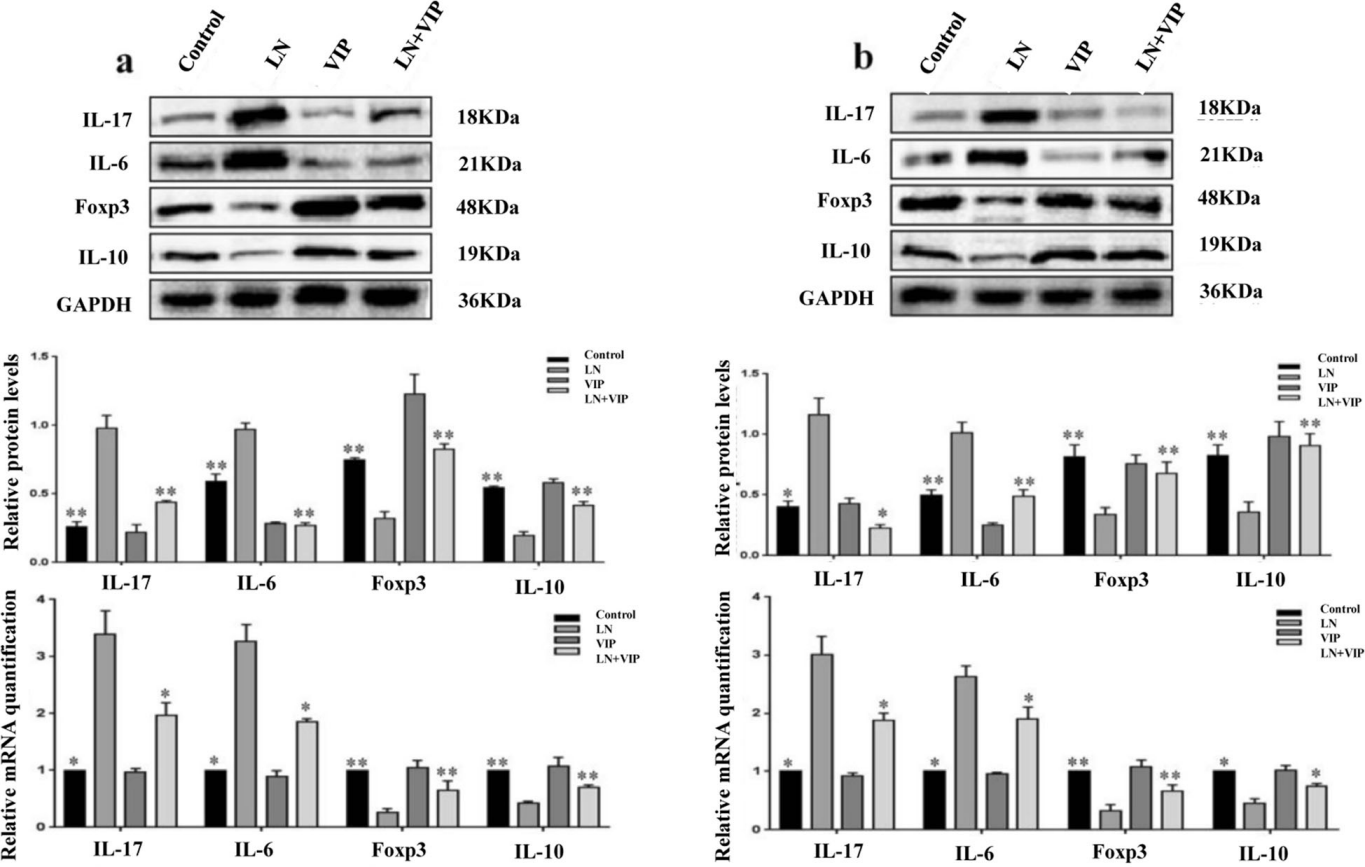
mRNA and protein expression levels of cytokines in renal tissue and lymphocytes. Total RNA and protein were extracted from the renal tissue and lymphocytes of mice. Then, the mRNA was quantified using real-time PCR, while the protein expression was evaluated using western blotting analysis. **a** mRNA and protein expression of IL-17, IL-6, Foxp3 and IL-10 in renal tissues and **b** mRNA and protein expression of IL-17, IL-6, Foxp3 and IL-10 in lymphocytes. *p < 0.05, **p < 0.01 compared with LN mice

IL-17, IL-6, Foxp3 and IL-10 mRNA and protein expression levels in renal tissues were analysed by qRT-PCR and western blotting. As shown in Fig. 6a, the mRNA and protein levels of IL-17 (3.39 ± 0.40 and 0.98 ± 0.09) and IL-6 (3.26 ± 0.29 and 0.96 ± 0.04) were higher, and the mRNA and protein levels of Foxp3 (0.25 ± 0.07 and 0.32 ± 0.04) and IL-10 (0.42 ± 0.03 and 0.19 ± 0.03) were lower in the renal tissues of LN mice than in those of control mice (IL-17: 1.0 ± 0.0 and 0.26 ± 0.03; IL-6: 1.0 ± 0.0 and 0.59 ± 0.05; Foxp3: 1.0 ± 0.0 and 0.74 ± 0.01 and IL-10: 1.0 ± 0.0 and 0.54 ± 0.01; *p* < 0.05 and *p* < 0.01). Compared to control treatment in LN mice, LN + VIP treatment significantly increased Foxp3 (0.65 ± 0.15 and 0.82 ± 0.03) and IL-10 (0.69 ± 0.04 and 0.41 ± 0.02) mRNA and protein expression and decreased IL-17 (1.97 ± 0.22, 0.43 ± 0.01) and IL-6 (1.84 ± 0.06, 0.26 ± 0.01) mRNA and protein expression. There were no significant differences between the control and VIP mice (*p* > 0.05).

### VIP regulated the gene and protein expression levels of pro- and anti-inflammatory cytokines in lymphocytes from LN mice

To assess the effects of VIP on the lymphocyte response in LN mice, the mRNA and protein levels of IL-17, IL-6, Foxp3 and IL-10 in lymphocytes were examined by qRT-PCR and western blotting (Fig. 6b). These results were consistent with the mRNA and protein expression data from renal tissue. mRNA and protein expression levels of IL-17 (3.01 ± 0.30, 1.15 ± 0.14) and IL-6 (2.62 ± 0.19, 1.01 ± 0.08) were higher, and mRNA and protein expression levels of Foxp3 (0.32 ± 0.11, 0.33 ± 0.05) and IL-10 (0.45 ± 0.07, 0.36 ± 0.07) were lower in lymphocytes from LN mice than in those from control mice (IL-17: 1.0 ± 0.0 and 0.39 ± 0.04; IL-6: 1.0 ± 0.0 and 0.49 ± 0.04; Foxp3: 1.0 ± 0.0 and 0.81 ± 0.09 and IL-10: 1.0 ± 0.0 and 0.82 ± 0.08; *p* < 0.05 and *p* < 0.01). Compared to control treatment in LN mice, LN + VIP treatment significantly increased Foxp3 (0.66 ± 0.10 and 0.67 ± 0.09) and IL-10 (0.74 ± 0.04 and 0.90 ± 0.99) mRNA and protein expression and decreased IL-17 (1.87 ± 0.12 and 0.22 ± 0.02) and IL-6 (1.89 ± 0.20 and 0.48 ± 0.05) mRNA and protein expression.

## Discussion

The present study revealed that VIP can restore and maintain the immune balance between Th17 and Treg cells both in vivo and in vitro by inhibiting Th17 cell differentiation and restoring the number of Treg cells. These effects significantly reduce autoantibody production and inhibit the expression of proinflammatory cytokines while stimulating the expression of anti-inflammatory cytokines. These effects also correlated with reductions in renal function defects and proteinuria, as well as the amelioration of renal pathology. Importantly, our findings suggested that VIP is similarly effective for regulating the Th17/Treg balance in the treatment of LN mice.

The distribution and function of T lymphocytes are some of the most important factors in the pathogenesis of autoimmune disorders. The pathogenesis of LN is a classical autoimmune disease characterized by T lymphocyte dysfunction, including Th17/Treg cell imbalance, which results in renal inflammation [19, 20]. In recent years, immune balance disruptions of Th17/Treg cell subsets have gradually become a research hotspot. Many of these studies have found that Th17 cells induce autoimmunity, whereas Treg cells inhibit these phenomena and maintain immune homeostasis. The results from our study indicate that the peripheral blood and spleen have increased numbers of Th17 cells but decreased numbers of Treg cells. These cell changes result in enhanced IL-17 and IL-6 expression but reduced Foxp3 and IL-10 expression in the renal tissues of LN mice, which are significant risk factors for the occurrence of renal inflammation and underlying causes for developing renal damage. Therefore, the regulation of Th17/Treg cell balance might be used to restore immune homeostasis to provide therapeutic benefits in autoimmune conditions.

VIP is a neuropeptide that is widely distributed in the body and is a potent anti-inflammatory factor that affects the regulation strategies of both the innate and adaptive immune systems [21, 22]. Therefore, it has emerged as a potential candidate for the treatment of various autoimmune and inflammatory diseases; VIP has also been shown to be effective in the prevention of autoimmune diseases, such as diabetes mellitus, rheumatoid arthritis, EAE, sepsis and inflammatory bowel disease [11, 23–27]. Gonzalez-Rey et al. [28] indicated that VIP participates in the maintenance of immune tolerance to ameliorate autoimmune disease progression by expanding the population of Treg cells. Subsequently, Deng et al. [29] found that VIP inhibits inflammatory responses in autoimmune diseases by upregulating Treg cells, but it has also been associated with downregulating Th17 cells. Thus, the therapeutic effect of VIP has been attributed to its capacity to downregulate inflammatory factors by switching the Th17/Treg balance. Here, we provide additional evidence that VIP administration in vivo and in vitro can inhibit Th17 cells and stimulate the Treg response, which ameliorates renal function defects in LN mice, while simultaneously improving the inflammatory state, autoantibody expression and renal damage.

This study enriched the understanding of the pathogenesis of LN and provided a theoretical basis for the application of VIP in LN. However, there are some limitations in present study. Any signalling pathways and transcription factors such as STAT3 and RORγt did not be explored. In order to explore the exact mechanism of VIP in LN, we are considering to detect some typical pathways or analyse the gene array in VIP-treated Th17 or Treg cells from lupus model to find out the differential signaling pathways and transcription factors, and even the interaction between them in the future study.

In summary, these experiments revealed that aberrant T-cell homeostasis is a crucial event in LN pathogenesis, and Th17/Treg imbalance appears to be an important key pathogenic player. This imbalance leads to increased serum levels of autoantibodies and renal levels of IL-6 and IL-17, as well as renal function defects. Furthermore, decreased renal levels of Foxp3 and IL-10 are associated with glomerulonephritis in pristane-induced lupus mice. VIP is reliable and effective for treating LN, and its therapeutic mechanisms should be effective for modulating Th17/Treg immune homeostasis by downregulating serum levels of autoantibodies and renal levels of IL-17 and IL-6 and upregulating renal levels of Foxp3 and IL-10, thus ameliorating renal function defects, proteinuria and renal damage.

## Conclusions

VIP is a potential therapeutic agent for treating LN and other abnormal Th17/Treg diseases in mice. Therefore, promising results concerning the therapeutic targeting of Th17/Treg cell balance may open new lines of investigation for LN treatment in the near future. However, additional research is needed to further explain this process.

## Data Availability

The datasets used and/or analysed during the current study are available from the corresponding author on reasonable request.

## Abbreviations

EAE: Experimental autoimmune encephalomyelitis
ESRD: End-stage renal disease
Foxp3: Forkhead box P3
IL-10: Interleukin-10
IL-17: Interleukin-17
IL-6: Interleukin-6
LN: Lupus nephritis
SLE: Systemic lupus erythaematosus
Th17 cells: T helper 17 cells
Treg cells: Regulatory T cells
VIP: Vasoactive intestinal peptide
